# Elastoplastic analytical solutions for a shallow-buried circular tunnel in a semi-infinite medium

**DOI:** 10.1371/journal.pone.0325194

**Published:** 2025-05-28

**Authors:** Tao Jiang, Lihui Xie, Chao Wang, Peiyuan He, Jinfeng Zou, Chunzhou Zhu, Fang Li

**Affiliations:** 1 Nanchang Rail Transit Group Co., Ltd., Nanchang, People’s Republic of China; 2 Metro Project Management Branch of Nanchang Rail Transit Group Limited Corporation, Nanchang, People’s Republic of China; 3 School of Civil Engineering, Central South University, Changsha, People’s Republic of China; 4 China Railway Shanghai Design Institute Group Co., Ltd., Shanghai, People’s Republic of China; 5 China Railway First Group Co., Ltd., Xi’an, People’s Republic of China; China Construction Fourth Engineering Division Corp. Ltd, CHINA

## Abstract

In order to explore the distribution pattern of the plastic zone around a shallow tunnel, the elastoplastic analytical solutions for a shallow-buried circular tunnel in a semi-infinite medium were proposed in this study to calculate the plastic zone radius and stresses around a shallow-buried circular tunnel. Based on the Hoek-Brown yield criterion, the solutions were derived by using the bipolar coordinate system method under the influence of the geotechnical gravity. The rationality of the proposed calculation method was verified by comparing with the existing calculation methods and numerical simulation methods, and the proposed method was further used to analyze the influence of the mechanical parameters of the rock and soil mass and uniform ground load on the plastic zone radius and stresses around a shallow-buried circular tunnel. The results show that the theoretical calculation results obtained by the proposed method fit better with the numerical simulation results, and the maximum and average errors between the two are 1.63% and 1.35%, respectively, so the calculation accuracy of the proposed method is proven to be high. The quality and strength of the geotechnical medium and the uniform ground load are both the main influencing factors of the plastic zone radius and stresses around a shallow-buried circular tunnel, and the plastic zone radius is negatively correlated with the quality and strength of the geotechnical medium, and the stresses are also negatively correlated with the quality and strength of the geotechnical medium, but are positively correlated with the uniform ground load. Therefore, the mechanical parameters of the rock and soil mass and uniform ground load should be controlled strictly to ensure the safety of shallow tunnels under construction.

## 1. Introduction

In view of the complexity of the mechanics of the surrounding rocks in a shallow-buried tunnel, the theoretical results on the mechanical properties around a shallow-buried tunnel have not yet reached a consensus. Since shallow buried tunnels are often excavated in shallow strata and close to the ground surface, it is easy to induce undesirable phenomena such as ground subsidence or even large-scale ground collapse during construction [1,2]. In order to ensure the safety of shallow tunnel construction, the mechanical properties of the rock and soil around shallow tunnels need to be investigated, and the calculation method for quantitatively characterizing the plastic zone radius, stresses, and other key parameters of the mechanics of the surrounding rock in shallow tunnels is further proposed to control the corresponding design and construction parameters in a reasonable range, so as to effectively avoid the geological and engineering disasters that may occur during the construction of shallow tunnels.

Tunnel excavation causes the original stress equilibrium in the surrounding rock to be broken, and a new stress equilibrium state is gradually formed. In the process of forming the new stress equilibrium, a part of the rock and soil mass around the tunnel enter the plastic yielding or even destruction state, so the plastic zone can be formed around the tunnel gradually. The plastic zone radius and stresses around the tunnel can affect the stability of the surrounding rock. Especially for shallow buried tunnels, due to their small burial depth, and the vault is closer to the ground surface, when the plastic zone extends to the ground surface, it will trigger a wide range of ground settlement, which is not conducive to the stability of the surrounding rock. In order to effectively improve the safety of shallow tunnel engineering construction, some in-depth studies have been carried out on the surrounding rock stresses in shallow tunnels. The research methods mainly include numerical simulation [3–6], experimental analysis [7–11] and theoretical derivation [12–19]. For the theoretical studies on the stress distribution pattern of the surrounding rock in shallow buried tunnels, Ai et al. [12] used the fractional calculus theory, Schwartz alternating method, and conformal mapping technique to establish an analytical method for the stress and displacement around shallow arbitrarily shaped tunnels excavated in viscoelastic transversely isotropic strata. Ai et al. [13] used the hybrid penalty function method, complex function method, and Schwartz alternating method to propose the stress solutions around shallow buried tunnels with arbitrary shapes in transversely isotropic rock mass. Ai et al. [14] adopted the Schwarz alternating method to obtain the solutions for the stresses of surrounding orthotropic rock with a shallow elliptical tunnel based on the solutions for the stresses of the transversely isotropic surrounding rock for deeply buried elliptical tunnels, and further developed a method for the stresses of the transversely isotropic surrounding rock for shallow elliptical tunnels. Ye and Ai [15] proposed a novel complex variable method for the stress and displacement fields around a shallow non-circular tunnel. Xu et al. [16] considered the influence of the seepage force and used the complex variable method to derive the explicit analytical solutions of seepage field and stress field of a shallow underwater tunnel excavated in an elastic rock mass. Fang et al. [17] proposed an analytical solution for the seepage-induced effective stresses around a shallow underwater tunnel in an elastic half plane. Wang et al. [18] considered the influence of the viscoelasticity of the ground, inclination of ground surface, effect of support and its delayed installation, and proposed a new method for predicting the time-dependent ground stresses and displacements induced by shallow tunnelling under initial stresses due to both gravity and surcharge loads. Wang et al. [19] derived the semi-analytical solutions for elastic and plastic stresses around a shallow circular tunnel based on the Mohr-Coulomb failure criterion. According to the above studies, the distribution pattern of the stresses around a shallow tunnel has been analyzed very thoroughly, which gives the basis of the elastic stresses for the solution of the elastic-plastic interface. However, the existing theoretical studies about the stresses of the surrounding rock in a shallow tunnel are mostly elastic solutions, the plastic solutions are still relatively few. In order to facilitate the solutions for the plastic zones around the shallow buried tunnels, the distribution pattern of the plastic stresses around the shallow buried tunnels still needs to be further explored.

Since the plastic zone is an important physical quantity describing the damage range of the surrounding geotechnical medium caused by tunnel excavation, it is an important research content in the stability analysis of the surrounding rock in shallow buried tunnels. At present, regarding the elastoplastic analysis for the rock and soil mass around shallow buried tunnels, some relevant studies have been carried out on the distribution pattern of the plastic zone around a shallow-buried tunnel. Massinas and Sakellariou [20] derived a closed-form solution for the problem of the plastic zone and stress distribution around a circular tunnel in an elastic-plastic half space by using bipolar coordinates, but the effect of gravity was ignored. Zareifard and Fahimifar [21] proposed a elasto-plastic analytical-numerical solution for a circular tunnel excavated in a strain-softening and Hoek Brown rock mass under the consideration of the axial-symmetry condition, but the explicit analytical solutions for the plastic zone are not given. Li et al. [22] used the complex variable method to propose the analytical solutions of the ground stresses and displacements of a shallow circular tunnel in an elastic half-plane under arbitrary distributed loads on ground surface. Zou et al. [23] considered the gravitational effect to derive the closed-form solutions for the stress distribution and plastic zone shape of the surrounding rock and the critical internal support pressure based on the bipolar coordinate system and Mohr-Coulomb failure criterion. Zeng et al. [24] considered the influence of the quasirectangular tunnel shapes, the ground surface, the tunnel depth, and the ground’s elastic/viscoelastic properties to propose analytical solutions for the mechanical mechanism of the stresses and displacements around the tunnels by using the Schwarz alternating method and complex variable method. Gao et al. [25] established a plane strain mechanical model for stress distribution around the holes in homogeneous elastoplastic media to further analyze the theories of rockburst based on butterfly-shaped plastic zones. Wang et al. [26] utilized the Schwartz alternating method to develop a new analytical approach for predicting the elastic stresses and displacements around the twin unequal circular tunnels, and gave the solutions for the plastic zone radius. Wang et al. [19] considered the influence of the unit weight of surrounding rock and the uniform surface surcharge pressure to propose an elastoplastic semi-analytical solution for the closed plastic zone around a shallow circular tunnel. According to the above investigation, although both the complex variable method and bipolar coordinate system method have been widely used to solve the plastic zone around shallow tunnels, the complex variable method is relatively complicated and often needs to be used in combination with the Schwarz alternating method, so an analytical expression for the distribution pattern of the plastic zone cannot be given directly. In contrast, the explicit analytical solutions for the plastic zone radius and stresses around a shallow buried tunnel can be obtained by the bipolar coordinate system method. For shallow tunnels with simple boundary shapes such as circles and ellipses, the bipolar coordinate system method is more effective.

Considering that shallow buried tunnels are different from deep buried tunnels [27–31], compared with deep buried tunnels, the center depth of shallow buried tunnels tends to be smaller, so the ratio of the center depth of shallow tunnels to the excavation radius should be satisfied to be less than 7 in this study. As we all know, the gravitational effect can be ignored when the mechanical analysis for the surrounding rock is carried out in deep buried tunnels [32–36]. However, the influence of the unit weight of surrounding rock cannot be ignored when the mechanical analysis for shallow buried tunnels [23,37–39]. In addition, unlike deep buried tunnels belonging to the full plane problem, shallow buried tunnels always belong to the semi-infinite plane problem. Therefore, the shallow tunnel is only symmetric about the tunnel centerline. Based on these, we consider the influence of the influence of the unit weight of surrounding rock, and further adopt the bipolar coordinate system method to develop an elastoplastic solution for the plastic zone radius and stresses around a shallow circular tunnel based on the Hoek-Brown yield criterion, which realizes the elastoplastic analysis for the surrounding rock of a shallow-buried circular tunnel. Therefore, elastoplastic analytical solutions for a shallow-buried circular tunnel in a semi-infinite medium are established, which provide theoretical guidance and reference for the design of similar shallow-buried tunnel projects.

## 2. Problem description and assumptions

**Fig 1:**
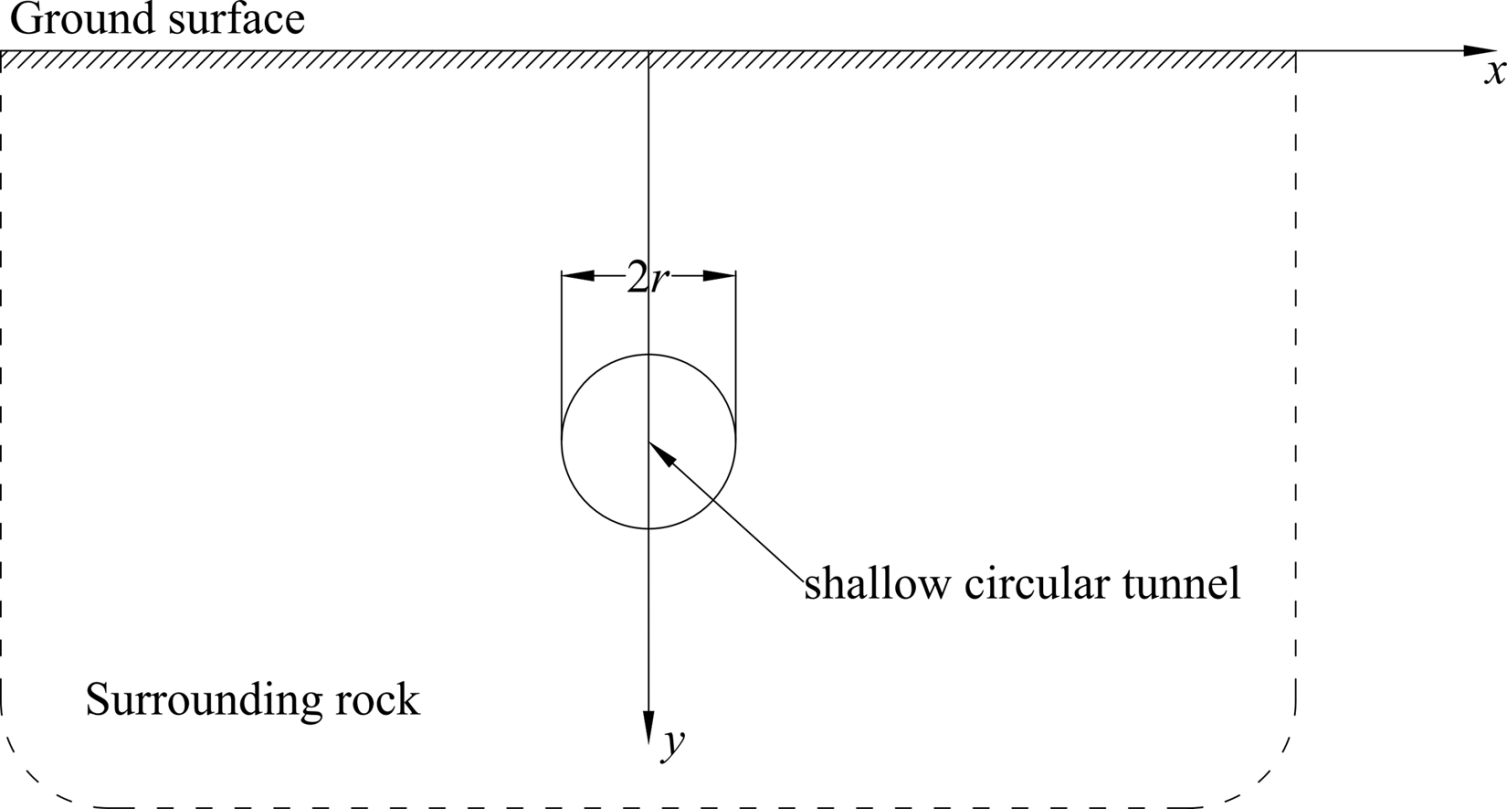
Schematic diagram of a shallow circular tunnel in the semi-infinite medium.

As shown in Fig 1, a shallow circular tunnel with radius *r* is the object of study in a semi-infinite medium. In the Cartesian coordinate system, the *x*-axis is represented as the ground. The *y*-axis crossing the center of the circular tunnel is the symmetrical axis of the tunnel and the semi-infinite medium. Besides, the tunnel axis burial depth is represented as *d*. Surrounding rock of the tunnel is assumed to be continuous, isotropous, and homogeneous. And the ratio of the tunnel burial depth to radius is not more than seven.

Before the excavation, the uniform ground load *P*_0_ acts on the *x*-axis, and the stress of the tunnel uniformly in a radial direction is also *P*_0_. Following the excavation, the internal support pressure is decreasing constantly until it decreases to a critical value *P*_*cr*_, and the surrounding rock in the shallow circular tunnel develops into the initial yield state. The plastic zone are extended around the tunnel with the internal support pressure less than the critical value *P*_*cr*_. In addition, the stress redistribution in the elastic zone can be ignored when the plastic zone is solved.

## 3. Methodology

### 3.1. Bipolar coordinate system

**Fig 2:**
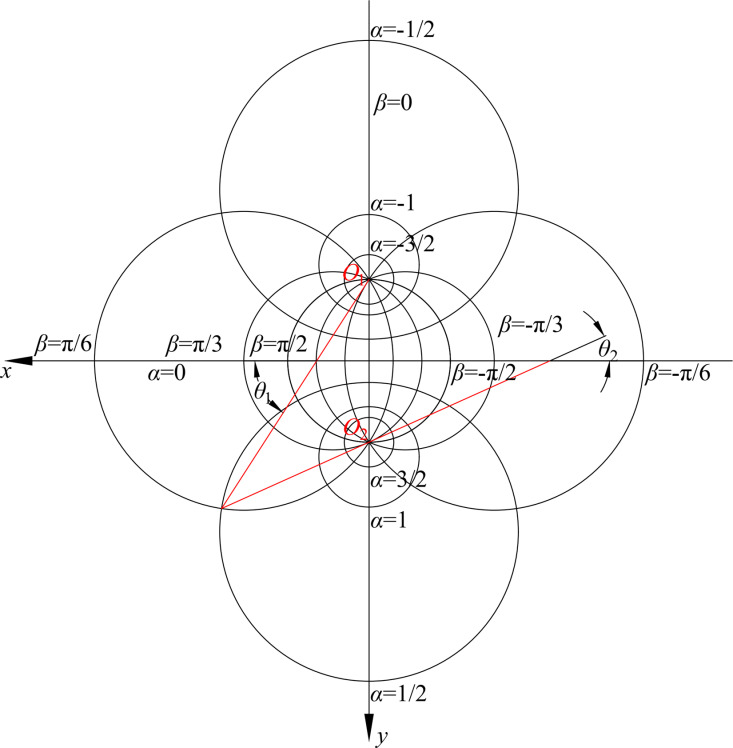
The bipolar coordinate system adopted in this study.

As shown in Fig 2, the two poles *O*_1_ and *O*_2_ are located on the *y*-axis and symmetric about the *x*-axis in the bipolar coordinate system. Each point in the bipolar coordinate system is determined by the intersection of two circles, and any point can be represented by the coordinates *α* and *β*. If *α* = 0, the curve is on the *x*-axis. And if *α* > 0, the circles lie below the *x*-axis and the center of the circles is located at the *y*-axis, conversely corresponding to negative values of *α* that lie above the *x*-axis. The function *β* is periodic with period 2π and can describe stresses and displacements, and the stresses and displacements will be continuous across *y*-axis. For the easy calculation, the coordinate *β* is set to change from -π to π while *β* = 0 on the *y*-axis.

The components of stress play important roles in solving the stress distribution and the plastic zone. They can be obtained by the stress function (*χ*) and the potential energy function of body-force (*Ω*). In the Cartesian coordinate system, considering the gravitational effect, the components of stress (*σ*_*x*_, *σ*_*y*_, and *τ*_*xy*_) can be solved by


$$\left\{ \begin{array}{c} \sigma_{x}=\frac{\partial^{2}\chi }{\partial y^{2}}+\Omega \\ \sigma_{y}=\frac{\partial^{2}\chi }{\partial x^{2}}+\Omega \\ \tau_{xy}=-\frac{\partial^{2}\chi }{\partial x\partial y} \end{array}\right.$$ (1)

According to Jeffery [40], the bipolar coordinate system (*α*, *β*) can be used to solve the plastic zone of surrounding rock, and the coordinate transformation technique between the Cartesian and bipolar coordinate systems is adopted, as shown in Eq. (2).


$$z=x+iy=ik\coth\frac{1}{2}\zeta$$ (2)

where *ζ* = *α* + i*β,* i is the imaginary unit, *k* is the distance between the origin and the pole in the bipolar coordinate system which can be expressed as follows:


$$k=\sqrt{d^{2}-r^{2}}$$ (3)

where *d* is the burial depth of the tunnel, and *r* is the radius of the tunnel.

In the bipolar coordinate system, *z* can be expressed as a function of *α* and *β*, as shown in Eq. (4).


$$z=ik\coth\frac{\left(\alpha +i\beta \right)}{2}$$ (4)

According to the Eq. (2), *ζ* can be expressed as a function of *z*, as shown in Eq. (5).


$$\zeta =\log\frac{z+ik}{z-ik}$$ (5)

The position vector (*z* + i*k*) is starting from point *O*_1_(0,-*k*) and ending at point *Z*(*x*,*y*), and it can be expressed by *r*_1_e^i*θ*1^ using the using the coordinate transformation technique [40]. Similarly, another position vector (*z*-i*k*) can be expressed by *r*_2_e^i*θ*2^. The expression of *z* with respect to *r*_1_, *r*_2_, *θ*_1_, *θ*_2_ can be obtained by substituting *r*_1_e^i*θ*1^ and *r*_2_e^i*θ*2^ into Eq. (4).


$$z=\log\frac{r_{1}}{r_{2}}+i(\theta_{1}-\theta_{2})$$ (6)

with


$$\left\{ \begin{array}{c} \alpha =\log(\frac{r_{1}}{r_{2}}\\ beta=\theta_{1}-\theta_{2} \end{array}\right.$$ (7)

In Fig 2, *θ*_1_ and *θ*_2_ are the angles between the radius *r*_1_ and *r*_2_ and the *x*-axis, respectively. *α* and *β* are constant. The curves are the circular arcs that pass through the poles *O*_1_ and *O*_2_. In addition, *r*_1_/*r*_2_ is the ratio of the modulus of complex numbers (*z* + i*k* and *z*-i*k*). Eq. (7) shows that the curves are a series of coaxial eccentric circles of radius *r* = *k*csch*α* with poles *O*_1_ and *O*_2_ as the limit points in the bipolar coordinates. If *α* < 0 and *r*_1_/*r*_2_ < 1, these circles surround the pole *O*_2_. And if *α* > 0 and *r*_1_/*r*_2_ > 1, these circles surround the pole *O*_1_. When passing through the *y*-axis connecting the poles, the coordinate *β* changes from −π to π. And if the function of *β* with a period of 2π can express the stress and strain, the stress and strain are continuous along the *y*-axis.

According to Jeffery [40], Eq. (8) can be obtained from Eq. (2) by the mathematical transformation and definition of conjugate function.


$$\mathrm{\backslash [}z=\alpha +i\beta =log\frac{x+i\left(y+k\right)}{x+i\left(y-k\right)}\mathrm{\backslash ]}$$ (8)

where *x* and *y* are the coordinates in the Cartesian system which can be obtained as follows:


$$\left\{ \begin{array}{c} x=\frac{k\sin\beta }{\cosh\alpha -\cos\beta }\\ y=\frac{k\sinh\alpha }{\cosh\alpha -\cos\beta } \end{array}\right.$$ (9)

Through any point *Z*, the arc length d*S*_*α*_ on the curve of *α* and the arc length d*S*_*β*_ on the curve of *β* can be expressed as


$$\left\{ \begin{array}{c} dS_{\alpha }=\sqrt{{\left(\frac{\partial x}{\partial \alpha }\right)}^{2}+{\left(\frac{\partial y}{\partial \alpha }\right)}^{2}}d\alpha =h_{\alpha }d\alpha \\ dS_{\beta }=\sqrt{{\left(\frac{\partial x}{\partial \beta }\right)}^{2}+{\left(\frac{\partial y}{\partial \beta }\right)}^{2}}d\beta =h_{\beta }d\beta \end{array}\right.$$ (10)

### 3.2. Hoek-Brown yield criterion

**Fig 3:**
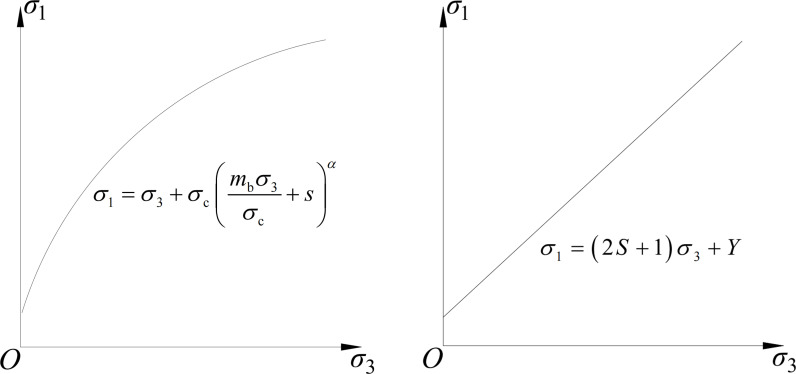
Comparison of Hoek-Brown and Mohr-Coulomb yield criteria: (a) Hoek-Brown yield criterion; (b) Mohr-Coulomb yield criterion.

The Hoek-Brown yield criterion is different from the Mohr-Coulomb yield criterion which can be shown in Fig 3.

The Hoek-Brown yield criterion is expressed as


$$\mathrm{\backslash [}\sigma_{\beta }=\sigma_{\alpha }+\sigma_{c}{\left(m_{b}\frac{\sigma_{\alpha }}{\sigma_{c}}+s\right)}^{a}\mathrm{\backslash ]}$$ (11)

where *σ*_*α*_ is the major principal stress, *σ*_*β*_ is the minor principal stress, *σ*_c_ is the lateral limitless compressive strength or uniaxial compressive strength of the intact rock and soil mass, and *m*_b_, *m*_*i*_, *α* and *s* are dimensionless constants which can be expressed as follows:


$$\mathrm{\backslash [}\left\{ \begin{array}{c} m_{b}=m_{i}\exp\left(\frac{GSI-100}{28-14D}\right)\\ s=\exp\left(\frac{GSI-100}{9-3D}\right)\\ \alpha =\frac{1}{2}+\frac{1}{6}\left(e^{-GSI/15}-e^{-20/3}\right) \end{array}\right.\mathrm{\backslash ]}$$ (12)

where *D* is the disturbance coefficient of the rock and soil mass. When *D* = 0, the rock mass is completely undisturbed. When *D* = 1, the rock mass is completely disturbed, especially for those severe blasting damage or strongly weathered rock masses. Since we take the shallow buried circular tunnel in the soft soil as the research object in this study, and the strength property of soft soil is similar to the strongly weathered or eroded rock mass, the disturbance coefficient of the rock and soil mass is set to 1 to better characterize the soft stratum around the shallow buried circular tunnel and reflect the severe disturbance effects of the shallow buried tunnel construction on the soft stratum.

### 3.3. Calculation model

**Fig 4:**
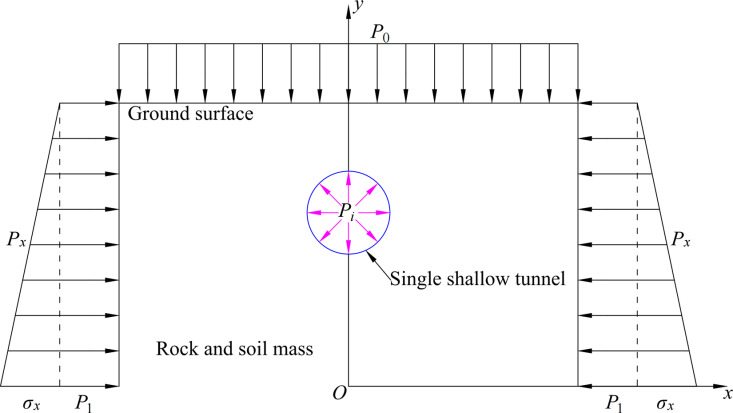
Mechanical calculation model for a single shallow circular tunnel.

Based on the bipolar coordinate system, considering the gravitational effect, the calculation model of the shallow circular tunnel in the semi-infinite medium is established in Fig 4. The tunnel burial depth *d* is *k*coth*α*_*i*_ and the tunnel radius *r* is *k*csh*α*_*i*_, where *k* = $\sqrt{\left(d^{2}-r^{2}\right)}$.

In the bipolar coordinate system, the components of stress can be expressed as follows:


$$\begin{array}{c} \left\{ \begin{array}{c} k\sigma_{\alpha }^{\prime }=\left[(\cosh\alpha -\cos\beta )\frac{\partial^{2}}{\partial \beta^{2}}-\sinh\alpha \frac{\partial }{\partial \alpha }-\sin\beta \frac{\partial }{\partial \beta }+\cosh\alpha \right]\left(\frac{\chi_{1}}{h}\right)\\ k\sigma_{\beta }^{\prime }=\left[(\cosh\alpha -\cos\beta )\frac{\partial^{2}}{\partial \alpha^{2}}-\sinh\alpha \frac{\partial }{\partial \alpha }-\sin\beta \frac{\partial }{\partial \beta }+\cos\beta \right]\left(\frac{\chi_{1}}{h}\right)\\ k\tau_{\alpha \beta }^{\prime }=-\left(\cosh\alpha -\cos\beta \right)\frac{\partial^{2}}{\partial \alpha \partial \beta }\left(\frac{\chi_{1}}{h}\right) \end{array}\right. \end{array}$$ (13)

### 3.4. Analysis of stress functions

If the gravitational effect is considered, in the bipolar coordinate system, the components of stress in the elastic zone can be expressed as follows:


$$\left\{ \begin{array}{c} k\sigma_{\alpha }^{\prime \prime }=\left[(\cosh\alpha -\cos\beta frac\partial^{2}\partial \beta^{2}-\sinh\alpha \frac{\partial }{\partial \alpha }-\sin\beta \frac{\partial }{\partial \beta }+\cosh\alpha \right]\left(\frac{\chi_{2}}{h}\right)+k\Omega \\ k\sigma_{\beta }^{\prime \prime }=\left[(\cosh\alpha -\cos\beta )\frac{\partial^{2}}{\partial \alpha^{2}}-\sinh\alpha \frac{\partial }{\partial \alpha }-\sin\beta \frac{\partial }{\partial \beta }+\cos\beta \right]\left(\frac{\chi_{2}}{h}\right)+k\Omega \end{array}\right.$$ (14)

If only the gravitational effect is considered, the body-force potential of the shallow circular tunnel in the semi-infinite medium can be expressed as follows:


\[Ω=−ωy\] (15)

Then, the formula for the components of stress in the plastic zone of the shallow circular tunnel in the semi-infinite medium can be expressed as follows:


$$\mathrm{\backslash [}\left\{ \begin{array}{c} \left(\cosh\alpha -\cos\beta \right)\left(\frac{\partial \sigma_{\alpha }}{\partial \alpha }+\frac{\partial \tau_{\alpha \beta }}{\partial \beta }\right)-\left(\sigma_{\alpha }\sinh\alpha +2\tau_{\alpha \beta }\sin\beta -\sigma_{\beta }\sinh\alpha \right)+f_{\alpha }=0\\ \left(\cosh\alpha -\cos\beta \right)\left(\frac{\partial \tau_{\alpha \beta }}{\partial \alpha }+\frac{\partial \sigma_{\beta }}{\partial \beta }\right)-\left(\sigma_{\beta }\sin\beta +2\tau_{\alpha \beta }\sinh\alpha -\sigma_{\alpha }\sin\beta \right)+f_{\beta }=0 \end{array}\right.\mathrm{\backslash ]}$$ (16)

where *f*_*α*_ and *f*_*β*_ can be respectively obtained as


$$f_{\alpha }=-\frac{\partial }{\partial \alpha }\left(\omega h\sinh\alpha \right)f_{\alpha }=-\frac{\partial }{\partial \beta }\left(\omega h\sinh\alpha \right)$$ (17)

### 3.5. Derivation of the analytical solutions for the elastic and plastic stress fields around a shallow-buried circular tunnel

#### 3.5.1. *Derivation of the stress field in the elastic zone of the surrounding rock.*

If the gravitational effect is not considered, the general rank form of the function of stress can be expressed as follows:


$$\frac{\chi_{1}}{h}=B_{0}\alpha (\cosh\alpha -\cos\beta )+(A_{1}\cosh2\alpha +B_{1}+C_{1}\sinh2\alpha cos\beta$$ (18)

And the function of stress which considers the gravitational effect can be expressed as follows:


$$\mathrm{\backslash [}\begin{array}{c} \frac{\chi_{2}}{h}=-\frac{1}{2}wk^{2}{csch\mathrm{\backslash nolimits}}^{2}\alpha_{i}\{\phi \sin\beta -\frac{1-2v}{2(1-v)}\alpha \sinh\alpha +\frac{5-6v}{2(1-v)}\coth\alpha_{i}(\cosh\alpha -\cos\beta )\alpha \\ -\frac{3-4v}{4(1-v)}(\cosh2\alpha -1)\cos\beta +\frac{1}{2}\left(\frac{5-6v}{2(1-v)}\coth\alpha_{i}-1\right)\sinh2\alpha \cos\beta \\ +\sum_{n=2}^{\infty }\frac{2\sinh\alpha_{i}e^{-n\alpha_{i}}}{\sinh^{2}n\alpha_{i}-n^{2}\sinh^{2}\alpha_{i}}\times [\sinh n\alpha_{i}\sinh n\alpha \sinh(\alpha -\alpha_{1})-n\sinh n\alpha_{i}\sinh n\alpha \sinh(\alpha -\alpha_{1})]\cos n\beta \} \end{array}\mathrm{\backslash ]}$$ (19)

Combining Eq. (18) with Eq. (19), the complete function of stress can be expressed as follows:


$$\mathrm{\backslash [}\begin{array}{c} \frac{\chi }{h}=\frac{\chi_{1}}{h}+\frac{\chi_{2}}{h}=B_{0}\alpha \left(\cosh\alpha -\cos\beta \right)+\left(A_{1}\cosh2\alpha +B_{1}+C_{1}\sinh2\alpha \right)\cos\beta -\frac{1}{2}wk^{2}{csch\mathrm{\backslash nolimits}}^{2}\alpha_{i}\{\phi \sin\beta -\frac{1-2v}{2(1-v)}\alpha \sinh\alpha \\ +\frac{5-6v}{2(1-v)}\coth\alpha_{i}(\cosh\alpha -\cos\beta )\alpha -\frac{3-4v}{4(1-v)}(\cosh2\alpha -1)\cos\beta +\frac{1}{2}\left(\frac{5-6v}{2(1-v)}\coth\alpha_{i}-1\right)\sinh2\alpha \cos\beta \\ +\sum_{n=2}^{\infty }\frac{2\sinh\alpha_{i}e^{-n\alpha_{i}}}{\sinh^{2}n\alpha_{i}-n^{2}\sinh^{2}\alpha_{i}}\times [\sinh n\alpha_{i}\sinh n\alpha \sinh(\alpha -\alpha_{1})-n\sinh n\alpha_{i}\sinh n\alpha \sinh(\alpha -\alpha_{1})]\cos n\beta \} \end{array}\mathrm{\backslash ]}$$ (20)

In summary, *σ*’‘_*α*_ and *σ*’‘_*β*_ can be obtained by jointing Eqs. (19) and (14). And the function of stress in the elastic zone can be obtained by


$$\mathrm{\backslash [}\left\{ \begin{array}{c} k\left(\sigma_{\alpha }^{\prime \prime }+\sigma_{\alpha }^{\prime }\right)=k\sigma_{\alpha }^{\prime \prime }+[B_{1}+A_{1}\cosh2\alpha +\left(C_{1}-\frac{1}{2}B_{0}\right)\sinh2\alpha \\ -\left(2A_{1}\sinh2\alpha +2C_{1}\cosh2\alpha -B_{0}\right)\sinh\alpha \cos\beta ]\\ k\left(\sigma_{\beta }^{\prime \prime }+\sigma_{\beta }^{\prime }\right)=k\sigma_{\beta }^{\prime \prime }+\{\left(\cosh\alpha -\cos\beta \right)\left[B_{0}\sinh\alpha +4\left(A_{1}\cosh2\alpha +C_{1}\sinh2\alpha \right)\cos\beta \right]+B_{1}\\ +A_{1}\cosh2\alpha +C_{1}\sinh2\alpha -2\sinh\alpha \cos\beta \left(A_{1}\sinh2\alpha +C_{1}\cos2\alpha \right)\} \end{array}\right.\mathrm{\backslash ]}$$ (21)

Here the compressive stress is specified as a positive value, and the tensile stress is negative. Then, the stresses in the elastic zone is expressed as follows:


$$\mathrm{\backslash [}\left\{ \begin{array}{c} \sigma_{\alpha }=-\frac{1}{k}[B_{1}+A_{1}\cosh2\alpha -(2A_{1}\sinh2\alpha \\ +2C_{1}\cosh2\alpha -B_{0})\sinh\alpha \cos\beta ]-\sigma_{\alpha }^{\prime \prime }\\ \sigma_{\beta }=-\frac{1}{k}\{(\cosh\alpha -\cos\beta )\left[B_{0}\sinh\alpha +4(A_{1}\cosh2\alpha +C_{1}\sinh2\alpha )\cos\beta \right]+B_{1}\\ +A_{1}\cosh2\alpha +C_{1}\sinh2\alpha -2\sinh\alpha \cos\beta (A_{1}\sinh2\alpha +C_{1}\cos2\alpha )\}-\sigma_{\beta }^{\prime \prime } \end{array}\right.\mathrm{\backslash ]}$$ (22)

Considering the boundary conditions without the gravitational effect, each constant of the function of stress can be expressed as follows:


$$\left\{ \begin{array}{c} M=(2\sinh^{3}\alpha_{i}{)}^{-1}\\ B_{0}=2kM(P_{i}-P_{0}cosh\alpha_{i}\\ A_{1}=-kM(P_{i}-P_{0})\sinh\alpha_{i}\\ B_{1}=kM\left[-P_{0}\cosh\alpha_{i}\sinh2\alpha_{i}+(P_{i}+P_{0})\sinh\alpha_{i}\right]\\ C_{1}=kM(P_{i}-P_{0})\cosh\alpha_{i} \end{array}\right.$$ (23)

#### 3.5.2. *Derivation of the stress field in the plastic zone of the surrounding rock.*

With the internal support pressure decreasing to the critical value, the plastic zone appears in the surrounding rock. By applying the critical condition, *σ*_*αi*_ and *σ*_*βi*_-*σ*_*αi*_ at the tunnel periphery are obtained from Eq. (22) and expressed as follows:


$$\mathrm{\backslash [}\left\{ \begin{array}{c} \sigma_{\alpha i}=-M\left[P_{0}\left(\sinh\alpha_{i}-\sinh2\alpha_{i}\cosh\alpha_{i}+\sinh\alpha_{i}\cosh2\alpha_{i}\right)+p_{cr}\left(\sinh\alpha_{i}-\sinh\alpha_{i}\cosh2\alpha_{i}\right)\right]-\sigma_{\alpha i}^{\prime \prime }\\ \sigma_{\beta i}-\sigma_{\alpha i}=-M\left(P_{cr}-P_{0}\right)\left(2\sinh2\alpha_{i}\cosh\alpha_{i}-4\sinh\alpha_{i}\cos^{2}\beta \right)-\left(\sigma_{\beta i}^{\prime \prime }-\sigma_{\alpha i}^{\prime \prime }\right) \end{array}\right.\mathrm{\backslash ]}$$ (24)

where *P*_cr_ is the function of *β* and can be obtained by substituting the second equation in Eq. (24) into Eq. (11).

With the internal support pressure decreasing to the critical value, the elastic-plastic interface will be created in the surrounding rock. Along the elastic-plastic interface, it is assumed that the principal stress obeys the Hoek-Brown yield criterion, and the stresses are continuous. Based on these, the components of stresses and the critical pressure that limits the extension of the plastic zone are satisfied as follows:


$$\sigma_{\alpha_{c}}^{e}=\sigma_{\alpha_{c}}^{p}=P_{c}$$ (25)

where *P*_c_ is the critical value that limits the extension of the plastic zone.

The stresses and the Hoek-Brown yield criterion on the elasto-plastic interface are expressed as follows:


$$\mathrm{\backslash [}\left\{ \begin{array}{c} \sigma_{\alpha ec}=-\frac{1}{k}[B_{1}+A_{1}\cosh2\alpha_{c}-(2A_{1}\sinh2\alpha_{c}\\ +2C_{1}\cosh2\alpha_{c}-B_{0})\sinh\alpha_{c}\cos\beta ]-\sigma_{\alpha ec}^{\prime \prime }\\ \sigma_{\beta ec}=-\frac{1}{k}\{\left(\cosh\alpha_{c}-\cos\beta \right)\left[B_{0}\sinh\alpha_{c}+4\left(A_{1}\cosh2\alpha_{c}+C_{1}\sinh2\alpha_{c}\right)\cos\beta \right]+B_{1}\\ +A_{1}\cosh2\alpha_{c}+C_{1}\sinh2\alpha_{c}-2\sinh\alpha_{c}\cos\beta \left(A_{1}\sinh2\alpha_{c}+C_{1}\cosh2\alpha_{c}\right)\}-\sigma_{\beta ec}^{\prime \prime }\\ \sigma_{\beta ec}-\sigma_{\alpha ec}=-\frac{1}{k}\{C_{1}\sinh2\alpha_{c}-B_{0}\sinh\alpha_{c}\cos\beta +\left(\cosh\alpha_{c}-\cos\beta \right)[B_{0}\sinh\alpha_{c}\\ +4\left(A_{1}\cosh2\alpha_{c}+C_{1}\sinh2\alpha_{c}\right)\cos\beta ]\}-\left(\sigma_{\beta ec}^{\prime \prime }-\sigma_{\alpha ec}^{\prime \prime }\right)\\ \sigma_{\beta ec}\text{=}\sigma_{\alpha ec}\text{+}\sigma_{ci}{\left(m_{b}\frac{\sigma_{\alpha ec}}{\sigma_{ci}}+s\right)}^{a} \end{array}\right.\mathrm{\backslash ]}$$ (26)

From Eq. (26), it can be seen that both *α*_*c*_ and *P*_*c*_ are variables with respect to *β*. *α*_*c*_ and *P*_*c*_ can be obtained by jointing Eqs. (25) and (26). However, the solution of *α*_*c*_ and *P*_*c*_ is too complicated to program with MATLAB to achieve.

Since the shear stress in the bipolar coordinate system is zero, Eq. (16) can be simplified as follows:


$$\mathrm{\backslash [}\left\{ \begin{array}{c} \left(\cosh\alpha -\cos\beta \right)\frac{\partial \sigma_{\alpha }}{\partial \alpha }-\left(\sigma_{\alpha }-\sigma_{\beta }\right)\sinh\alpha -\frac{\partial }{\partial \alpha }\left(\omega h\sinh\alpha \right)=0\\ \left(\cosh\alpha -\cos\beta \right)\frac{\partial \sigma_{\beta }}{\partial \beta }-\left(\sigma_{\beta }-\sigma_{\alpha }\right)\sin\beta -\frac{\partial }{\partial \beta }\left(\omega h\sinh\alpha \right)=0 \end{array}\right.\mathrm{\backslash ]}$$ (27)

Combining Eq. (11) with Eq. (27), the expression of *σ*_*α*_ can be obtained as follows:


$$\mathrm{\backslash [}\frac{\partial \sigma_{\alpha }}{\partial \alpha }+\frac{\sigma_{c}\sinh\alpha }{\cosh\alpha -\cos\beta }{\left(m_{b}\frac{\sigma_{\alpha }}{\sigma_{c}}+s\right)}^{a}+\frac{k\omega \sinh^{2}\alpha }{{\left(\cosh\alpha -\cos\beta \right)}^{3}}-\frac{k\omega \cosh\alpha }{{\left(\cosh\alpha -\cos\beta \right)}^{2}}=0\mathrm{\backslash ]}$$ (28)

**Fig 5:**
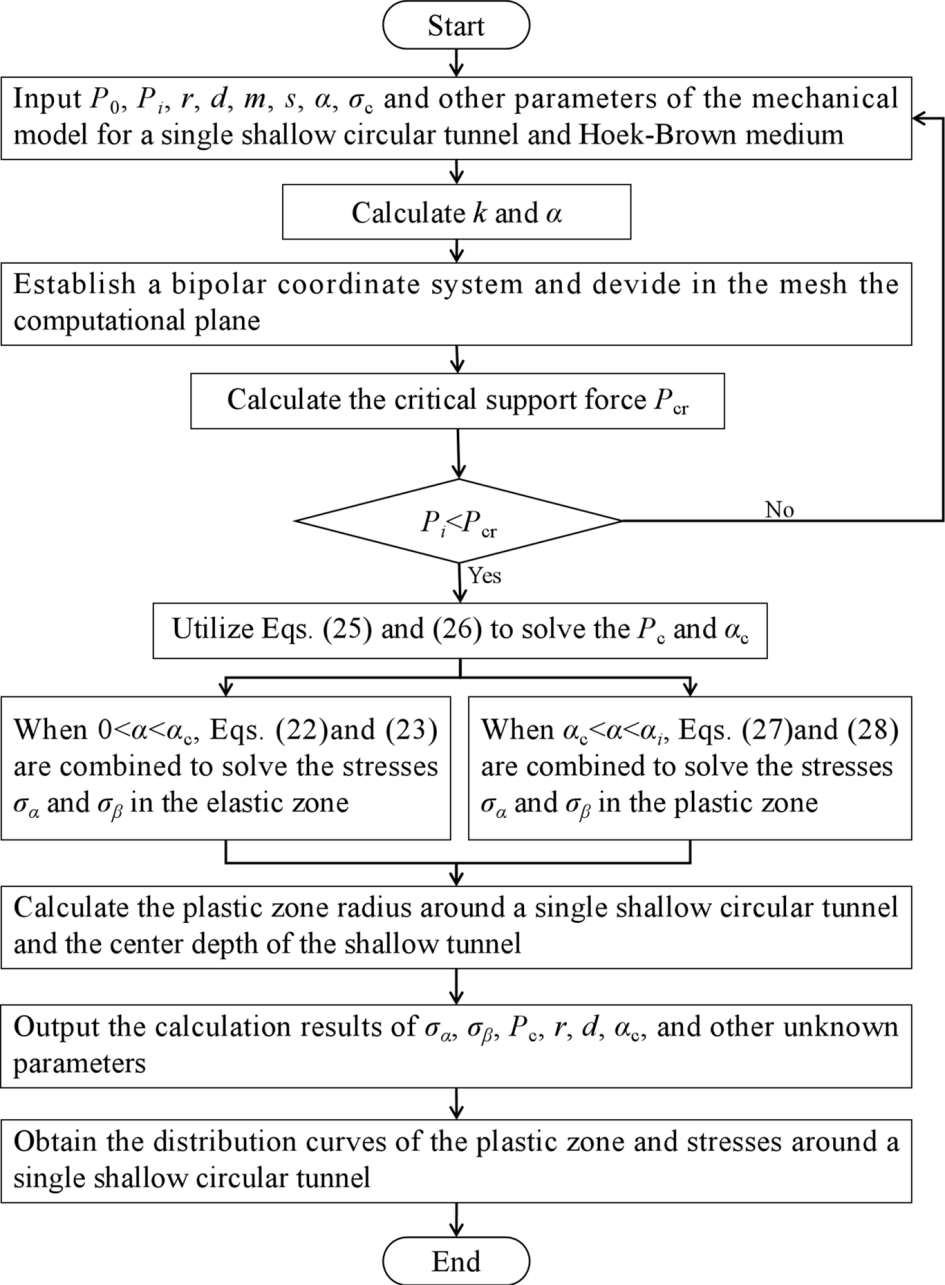
Calculation flowchart of the method proposed in this study.

By substituting *σ*_*α*_ into Eq. (11), *σ*_*β*_ can be obtained. Therefore, the above derivation for the elastoplastic analytical solutions for a shallow-buried circular tunnel in a semi-infinite medium can be calculated by the following flow chart, as shown in Fig 5.

## 4. Validation

**Fig 6:**
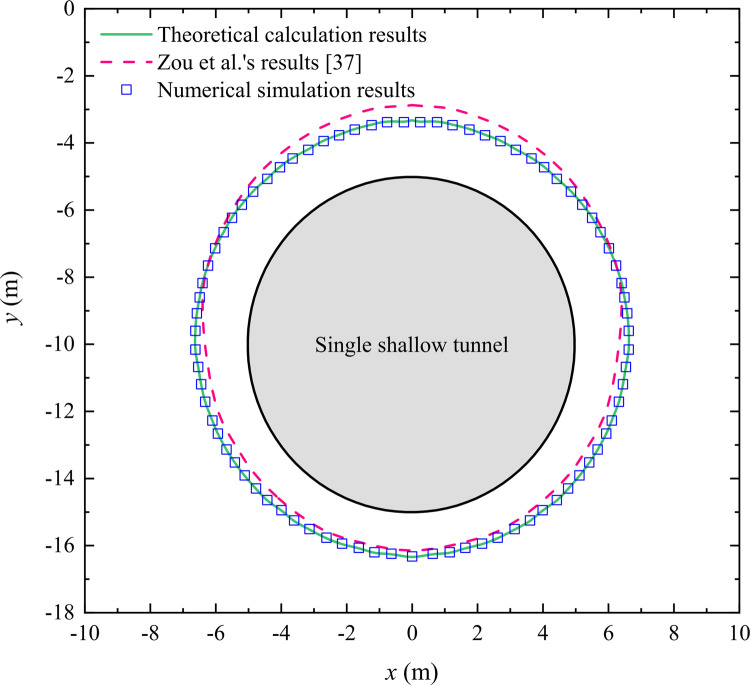
Comparison for the results of the plastic zone radius around a shallow tunnel obtained by the different methods.

**Fig 7:**
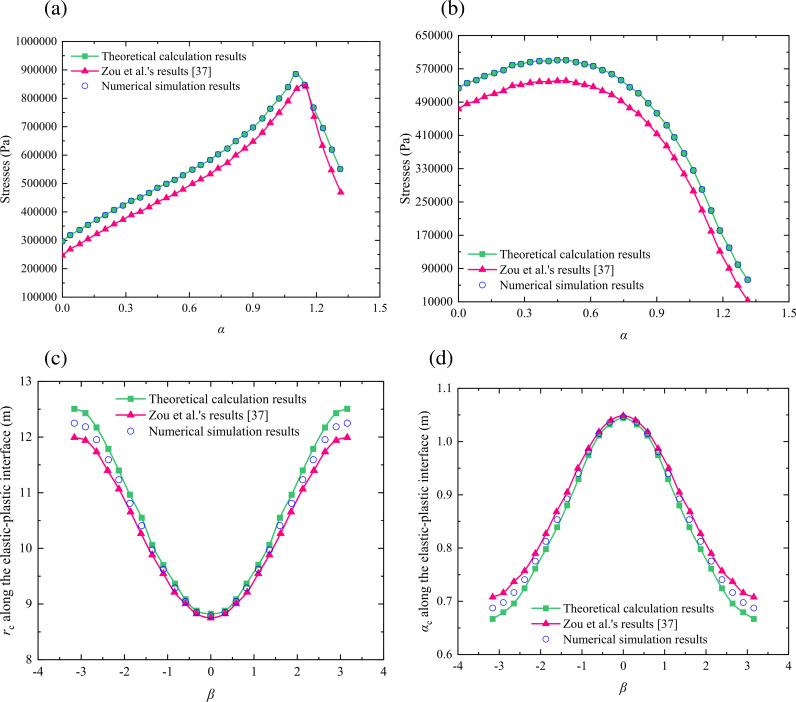
Comparison for the results of the mechanical parameters around a shallow tunnel based on the Hoek-Brown and Mohr-Coulomb criteria: (a) stress *σ*_*β*_; (b) stress *σ*_*α*_; (c) *r*_c_ along the elastic-plastic interface; (d) *α*_c_ along the elastic-plastic interface.

In order to verify the rationality of the calculation method proposed in this study, we compare the proposed method with Zou et al.’s method [23] and the numerical simulation method, respectively. Based on the calculation case described in the literature [23], we establish a computational example for verifying the rationality of the proposed method, which is shown in Tables 1 and 2. The calculation results of the plastic zone radius and principal stresses in the surrounding rock obtained by the above different methods are compared, respectively, and the results are shown in Figs 6 and 7.

**Table 1:** Parameters of the Hoek-Brown and Mohr-Coulomb yield criteria.

Hoek-Brown criterion			Mohr-Coulomb criterion	
*m*_b_	*a*	*S*	*c* (kPa)	*φ* (°)
0.5514	0.544	0.000138	60	25

**Table 2:** Parameters of the surrounding rock.

*P*_0_ (kPa)	*P*_*i*_ (kPa)	*r* (m)	*d* (m)	*σ*_c_ (kPa)	*v*
250	50	5	10	5	0.3

Fig 6 illustrates the distribution pattern of the plastic zone around a shallow tunnel. Compared with the results obtained by Zou et al. [23], the theoretical values obtained by the proposed method are closer to the numerical simulation results. The maximum and average errors between the theoretical calculation results and the numerical simulation results are 1.63% and 1.35%, and the maximum and average errors between Zou et al.’s results and the numerical simulation results are 15.12% and 4.32%. Obviously, the theoretical calculation results fit better with the numerical simulation results, and the calculation accuracy of the proposed method is higher. Considering the Mohr-Coulomb yield criterion used by Zou et al. in the literature [23], when the shallow tunnel is close to the ground, the extent of plastic zone around the shallow tunnel will gradually deviate from the tunnel center, which confirms the rationality of Zou et al.’s study results.

Fig 7 describes the distribution pattern of the stresses around a shallow tunnel. For the stress *σ*_*β*_, Fig 7(a) shows that the maximum and average errors between the theoretical calculation results and the numerical simulation results are 1.45% and 1.06%, and the maximum and average errors between Zou et al.’s results and the numerical simulation results are 16.77% and 4.79%. For the stress *σ*_*α*_, Fig 7(b) shows that the maximum and average errors between the theoretical calculation results and the numerical simulation results are 1.78% and 1.25%, and the maximum and average errors between Zou et al.’s results and the numerical simulation results are 18.49% and 5.71%. For the *r*_*c*_ along the elastic-plastic interface, Fig 7(c) shows that the maximum and average errors between the theoretical calculation results and the numerical simulation results are 1.38% and 0.67%, and the maximum and average errors between Zou et al.’s results and the numerical simulation results are 2.10% and 1.23%. For the *α*_*c*_ along the elastic-plastic interface, Fig 7(d) shows that the maximum and average errors between the theoretical calculation results and the numerical simulation results are 1.46% and 0.72%, and the maximum and average errors between Zou et al.’s results and the numerical simulation results are 2.98% and 1.42%. Therefore, compared with the results obtained by Zou et al. [23], the theoretical values obtained by the proposed method fit better with the numerical simulation results, which further verifies that the calculation accuracy of the proposed method is higher. In addition, when the rocks around a shallow tunnel are yielded to cause the plastic zone formed, the stresses in the surrounding rock under the Hoek-Brown yield criterion is more than them under the Mohr-Coulomb yield criterion. But in the plastic zone, the growing trend for the minor principal stresses based on two kinds of the yield criteria are nearly identical [37,39,41,42]. By observing the major principal stresses, we can see that the creation of the plastic zone under the Hoek-Brown yield criterion is earlier than it under the Mohr-coulomb yield criterion. When the plastic zone is just formed, the values of the major principal stresses based on the Hoek-Brown yield criterion are slightly less than them based on the Mohr-coulomb yield criterion. However, the former are gradually more than the latter, and the average errors between the two are always less than 8.5%, which is less than the 20% error limit allowed by engineering experience. Therefore, it confirms the rationality of the calculation method proposed in this study effectively.

## 5. Parameter analysis for the influencing factors of the surrounding rock in a shallow-buried circular tunnel

### 5.1. Discussion for the influence of the mechanical parameters of the rock and soil mass

**Fig 8:**
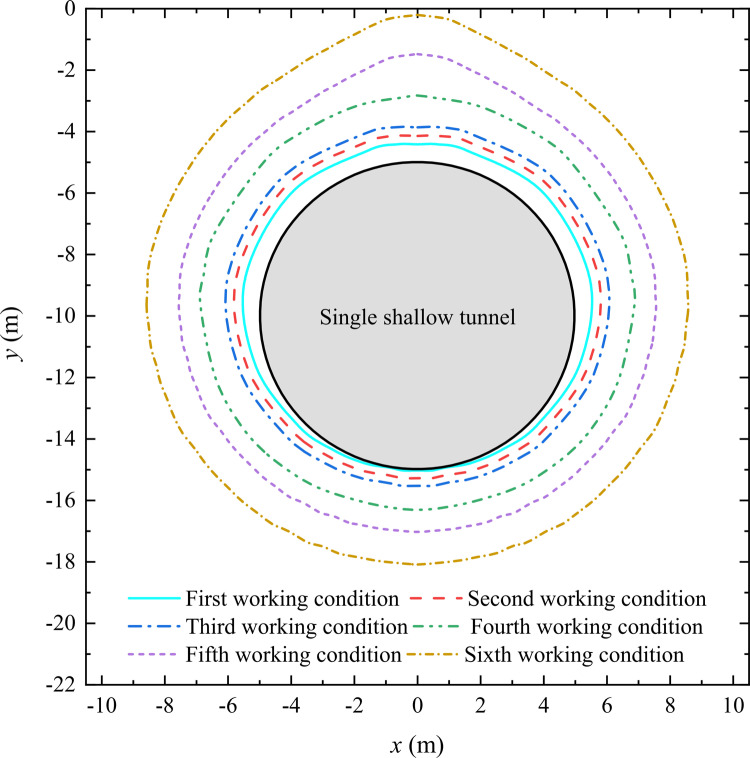
Distribution pattern of the plastic zone radius around a shallow-buried circular tunnel under different working conditions.

**Fig 9:**
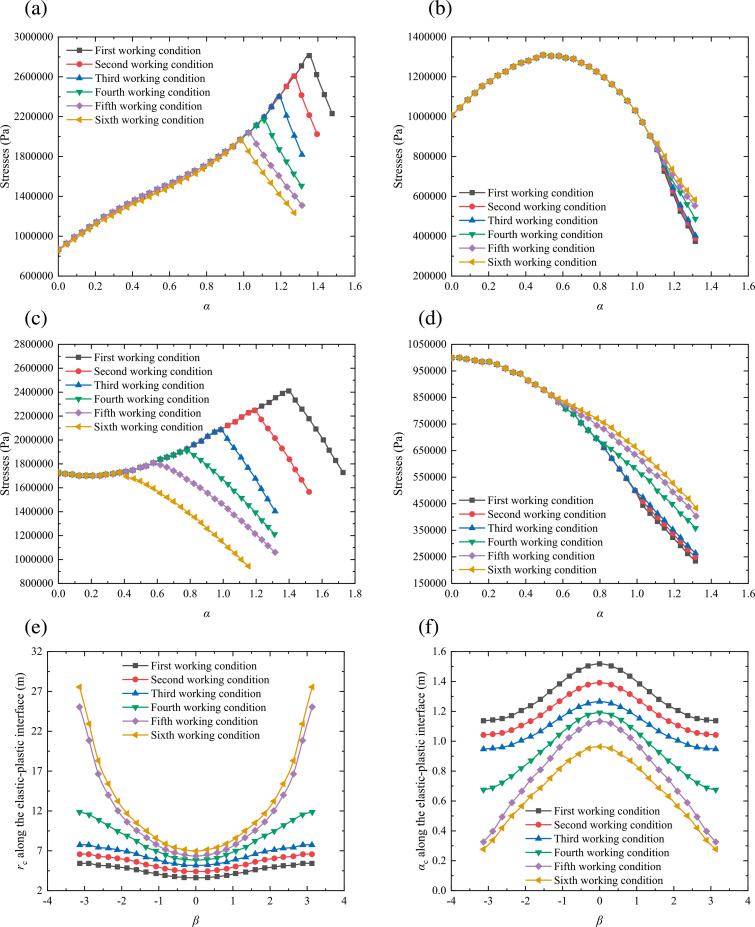
Distribution pattern of the mechanical parameters around a shallow-buried circular tunnel under different working conditions: (a) stress *σ*_*β*_ with *β* = π/4; (b) stress *σ*_*α*_ with *β* = π/4; (c) stress *σ*_*β*_ with *β* = 3π/4; (d) stress *σ*_*α*_ with *β* = 3π/4; (e) *r*_c_ along the elastic-plastic interface; (f) *α*_c_ along the elastic-plastic interface.

For analyzing the influence of the rock and soil mass, we utilize the control variable method to keep the *d*, *r*, *P*_0_, *v* and other basic parameters of the shallow-buried circular tunnel constant, which are shown in Table 3. The mechanical parameters of the rock and soil mass are set in Table 4, which are corresponding to different working conditions. According to Tables 3 and 4, the proposed method is used to calculate the distribution patterns of the plastic zone radius and stresses around a shallow-buried circular tunnel, and the analytical results are shown in Figs 8 and 9, respectively.

**Table 3:** Basic parameters of a single shallow tunnel.

Parameters	Values
Tunnel burial depth *d* (m)	10
Tunnel radius *r* (m)	5
Uniform ground load *P*_0_ (MPa)	1
Poisson’s ratio *v*	0.3

**Table 4:** Mechanical parameters of the rock and soil mass under different working conditions.

Working conditions	Parameters of the rock and soil mass								
	*r* (m)	*P*_0_ (MPa)	*σ*_c_ (MPa)	*GSI*	*m*_*i*_	*m*_b_	*S*	*a*	*v*
1	5	1	5	50	16	0.4499	0.00024	0.51	0.3
2	5	1	5	40	15.6	0.2147	0	0.51	0.3
3	5	1	5	30	12	0.0809	0	0.5	0.3
4	5	1	5	20	9.6	0.0317	0	0.55	0.3
5	5	1	5	10	10	0.0161	0	0.6	0.3
6	5	1	5	5	10	0.0113	0	0.62	0.3

Fig 8 demonstrates the distribution pattern of the plastic zone radius around a shallow-buried circular tunnel under the influence of the mechanical parameters of the rock and soil mass. As we all know, the quality and strength of the geotechnical medium are determined by the mechanical parameters of the rock and soil mass, so the quality and strength of the geotechnical medium are the influencing factors of the surrounding rock in a shallow-buried circular tunnel. From Fig 8, the plastic zone radius gradually increases with *GSI* decreasing, and the two are negatively correlated. Based on the Hoek-Brown yield criterion, *GSI* is larger, and the quality and strength of the geotechnical medium are better. Therefore, the quality and strength of the geotechnical medium are smaller, and the distribution range of the plastic zone is larger. The stability of the surrounding rock during tunnel construction is also relatively poor. Besides, Fig 8 also reflects that the extent of the plastic zone increases as the rock strength decreasing. Especially in some rocks with poor quality, the extent at the top of the plastic zone has a tendency to expand rapidly as the rock strength decreasing. This phenomenon is consistent with the situation in actual engineering [17,43,44].

Figs 9(a) and (d) shows that the creation of the plastic zone around a shallow-buried circular tunnel is advanced as the rock and soil mass decreasing. The major principal stresses of the plastic zone are negatively correlated with the strength of the rock and soil mass, and the minor principal stresses of the plastic zone are positively correlated with the strength of the rock and soil mass. Figs 9(e) and (f) indicates that the critical stresses along the elastic-plastic interface decrease gradually with the strength of the rock and soil mass increasing. Therefore, the quality and strength of the geotechnical medium is an important influencing factor for the surrounding rock in a shallow-buried circular tunnel, and we should pay attention to the influence of the mechanical parameters of the rock and soil mass on the plastic zone radius and stresses around a shallow-buried circular tunnel to guarantee the safety of tunnel engineering construction.

### 5.2. Discussion for the influence of the uniform ground load

**Fig 10:**
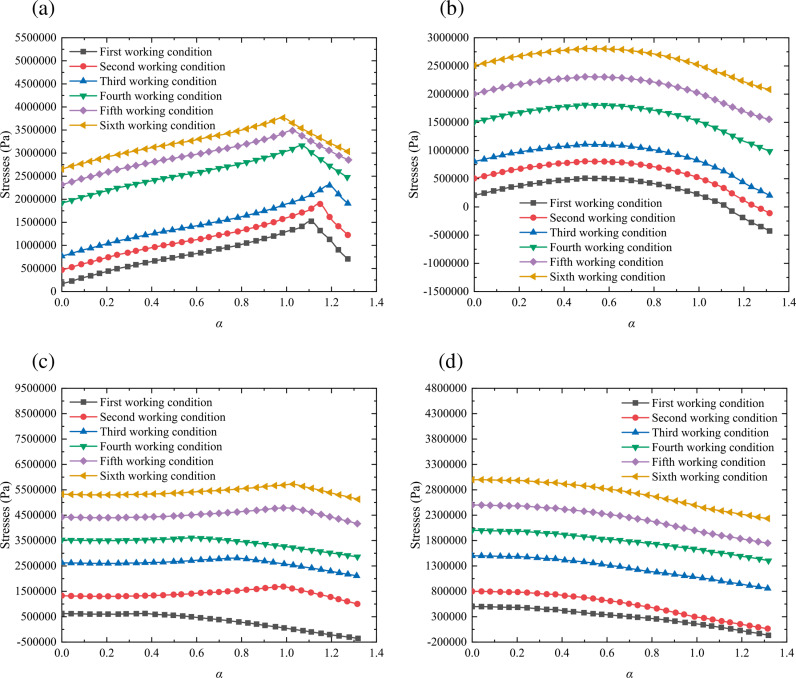
Distribution pattern of the stresses in the surrounding rock under different working conditions: (a) stress *σ*_*β*_ with *β* = π/4; (b) stress *σ*_*α*_ with *β* = π/4; (c) stress *σ*_*β*_ with *β* = 3π/4; (d) stress *σ*_*α*_ with *β* = 3π/4.

For analyzing the influence of the uniform ground load, we also use the control variable method to keep the uniform ground load *P*_0_ as a single variable, and set the uniform ground load *P*_0_ as 400kPa, 800kPa, 1500kPa, 2000kPa, 2500kPa, and 3000kPa, respectively. The different working conditions are shown in Table 5, and the proposed method is used to calculate the distribution patterns of the stresses around a shallow-buried circular tunnel under these working conditions. The corresponding results are shown in Fig 10.

**Table 5:** Uniform ground load under different working conditions.

Working conditions	Uniform ground load	Parameters of the tunnel			Parameters of the surrounding rock						
	*P*_0_ (kPa)	*P*_*i*_ (MPa)	*r* (m)	*d* (m)	*σ*_c_ (MPa)	*v*	*GSI*	*m*_*i*_	*m*_b_	*S*	*a*
1	400	0.2	5	10	5	0.3	20	9.6	0.551	0	0.55
2	800	0.2	5	10	5	0.3	20	9.6	0.551	0	0.55
3	1500	0.2	5	10	5	0.3	20	9.6	0.551	0	0.55
4	2000	0.2	5	10	5	0.3	20	9.6	0.551	0	0.55
5	2500	0.2	5	10	5	0.3	20	9.6	0.551	0	0.55
6	3000	0.2	5	10	5	0.3	20	9.6	0.551	0	0.55

Fig 10 demonstrates the distribution pattern of the stresses in the surrounding rock under the influence of the uniform ground load. The major and minor principal stresses around a shallow tunnel in the surrounding rock increases as the uniform ground load increasing, and the two are positively correlated. However, the rate of growth is decreasing, so the generation rate of the stresses around a shallow tunnel is negatively correlated with the uniform ground load. Influenced by this, we should set appropriate construction parameters to control the stresses around shallow tunnels in a reasonable range to reduce the adverse effects of plastic zones and ensure the safety of tunnel construction.

## 6. Limitations

According to the basic assumptions, derivations and discussions in this study, the elastoplastic analytical solutions for a shallow-buried circular tunnel in a semi-infinite medium have several limitations as follows:

(1) The elastoplastic analytical solutions for a shallow-buried circular tunnel in a semi-infinite medium are achieved by the method proposed in this study. Compared with the finite element methods, the functional characterization for the plastic zone radius and stresses around a shallow-buried circular tunnel can be achieved by the obtained elastoplastic analytical solutions, and the computational efficiency is effectively improved, but they are not applicable to shallow tunnels under construction in composite strata. While the existing finite element methods can provide the solutions for shallow tunnels under construction in composite strata by refining the model and stratifying geological layers. In order to better enhance the engineering applicability of the proposed method, the computational program established in this study should be embedded in the finite element software to realize the solution for a shallow-buried circular tunnel in composite strata in the subsequent research, and the accurate numerical solution for a shallow-buried circular tunnel in the corresponding state should be given.
(2) Tunnel shapes are also an important factor affecting the distribution of plastic zone. Based on the elastoplastic analytical solutions for a shallow-buried circular tunnel in this study, we should further analyze the distribution pattern of the plastic zone radius and stresses around a shallow tunnel with the horseshoe, portal, rectangular, and other common shapes. Based on the Hoek-Brown yield criterion, the elastoplastic solutions for a shallow tunnel with arbitrarily shaped should be proposed to enhance the scope of engineering applicability of the method proposed in this study.

## 7. Conclusions

Based on the bipolar coordinate system, a novel elastoplastic analytical solution was proposed to determine the plastic radius, shape of the plastic zone, and stress distribution for the shallow circular surrounding rock burred in a semi-infinite medium for the first time. The gravity effect and the characteristics of weak rocks and nonlinear were also incorporated. Compared to existing solutions, the innovations of this study are as follows:

(1) The proposed elastoplastic analytical solution can not only solve the plastic radius around a shallow circular tunnel but also determine the shape of the plastic zone and the stress distribution in the surrounding rock.
(2) The shape of the plastic zone around the shallow buried circular tunnel is approximately circular, but as the size of the plastic zone gradually increases, it gradually presents an upwardly convex teardrop shape. Therefore, the distribution range of the plastic zone affects the shape of the plastic zone, which tends to be connected upward with the ground surface as the plastic zone expands.
(3) The quality and strength of the geotechnical medium and the uniform ground load are both the main influencing factors of the plastic zone radius and stresses around a shallow-buried circular tunnel. The mechanical parameters of the rock and soil mass as the key factor to decide on the quality and strength of the geotechnical medium, with *GSI* decreasing, the strength of the rock and soil mass decreases correspondingly, and both of the plastic zone radius and minor principal stresses of the plastic zone gradually increase, but the major principal stresses of the plastic zone gradually decreases. The critical stresses along the elastic-plastic interface decrease gradually with the strength of the rock and soil mass increasing. Besides, with the uniform ground load increasing, the major and minor principal stresses both increase, but their growth rate is decreasing. Therefore, the distribution range of the plastic zone around a shallow-buried circular tunnel is negatively correlated with the quality and strength of the geotechnical medium, and the stresses around a shallow-buried circular tunnel is negatively correlated with the quality and strength of the geotechnical medium, but is positively correlated with the uniform ground load. In the actual project, the above influencing factors should be controlled within appropriate limits by adjusting the design and construction parameters to ensure the safety of shallow tunnel construction.
